# Use of nCounter mRNA profiling to identify at-arrival gene expression patterns for predicting bovine respiratory disease in beef cattle

**DOI:** 10.1186/s12917-022-03178-8

**Published:** 2022-02-23

**Authors:** Matthew A. Scott, Amelia R. Woolums, Cyprianna E. Swiderski, Alexis C. Thompson, Andy D. Perkins, Bindu Nanduri, Brandi B. Karisch, Dan R. Goehl

**Affiliations:** 1 Veterinary Education, Research, and Outreach Center, Texas A&M University and West Texas A&M University, Canyon, TX 79015 USA; 2 Department of Pathobiology and Population Medicine, Mississippi State University, Starkville, MS 39762 USA; 3 School of Animal and Comparative Biomedical Sciences, University of Arizona, Tucson, AZ 85721 USA; 4 Department of Computer Science and Engineering, Mississippi State University, Starkville, MS 39762 USA; 5 Department of Comparative Biomedical Sciences, Mississippi State University, Starkville, MS 39762 USA; 6 Department of Animal and Dairy Sciences, Mississippi State University, Starkville, MS 39762 USA; 7 Professional Beef Services, LLC, Canton, MO 63435 USA

**Keywords:** Bovine respiratory disease, NanoString, mRNA, Gene expression, Biomarkers, Disease prediction, Infectious disease, Host immunology, Beef cattle, Bioinformatics

## Abstract

**Background:**

Transcriptomics has identified at-arrival differentially expressed genes associated with bovine respiratory disease (BRD) development; however, their use as prediction molecules necessitates further evaluation. Therefore, we aimed to selectively analyze and corroborate at-arrival mRNA expression from multiple independent populations of beef cattle. In a nested case-control study, we evaluated the expression of 56 mRNA molecules from at-arrival blood samples of 234 cattle across seven populations via NanoString nCounter gene expression profiling. Analysis of mRNA was performed with nSolver Advanced Analysis software (*p* < 0.05), comparing cattle groups based on the diagnosis of clinical BRD within 28 days of facility arrival (*n* = 115 Healthy; *n* = 119 BRD); BRD was further stratified for severity based on frequency of treatment and/or mortality (Treated_1, *n* = 89; Treated_2+, *n* = 30). Gene expression homogeneity of variance, receiver operator characteristic (ROC) curve, and decision tree analyses were performed between severity cohorts.

**Results:**

Increased expression of mRNAs involved in specialized pro-resolving mediator synthesis (*ALOX15*, *HPGD*), leukocyte differentiation (*LOC100297044*, *GCSAML*, *KLF17*), and antimicrobial peptide production (*CATHL3*, *GZMB*, *LTF*) were identified in Healthy cattle. BRD cattle possessed increased expression of *CFB,* and mRNA related to granulocytic processes (*DSG1*, *LRG1*, *MCF2L*) and type-I interferon activity (*HERC6, IFI6, ISG15, MX1*). Healthy and Treated_1 cattle were similar in terms of gene expression, while Treated_2+ cattle were the most distinct. ROC cutoffs were used to generate an at-arrival treatment decision tree, which classified 90% of Treated_2+ individuals.

**Conclusions:**

Increased expression of complement factor B, pro-inflammatory, and type I interferon-associated mRNA hallmark the at-arrival expression patterns of cattle that develop severe clinical BRD. Here, we corroborate at-arrival mRNA markers identified in previous transcriptome studies and generate a prediction model to be evaluated in future studies. Further research is necessary to evaluate these expression patterns in a prospective manner.

**Supplementary Information:**

The online version contains supplementary material available at 10.1186/s12917-022-03178-8.

## Background

Bovine respiratory disease (BRD) is the leading clinical disease in post-weaned beef production systems throughout North America [1–3]. BRD is a multifactorial disease that culminates from complex interactions between viral and bacterial etiologies, the host, and adverse environmental circumstances, such as novel housing and feeding situations [3–7]. While novel diagnostic and therapeutic programs have been developed over the past decade, rates of morbidity and mortality related to BRD remains relatively unchanged [2, 8–10]. Ultimately, antemortem diagnosis of BRD is imprecise, and no gold standard modality currently exists for determining real-time therapeutic intervention [11–14]. Subsequently, at-arrival mass medication of cattle with antimicrobials (e.g., metaphylaxis) is commonly employed to improve cattle herd health and long-term economic performance [15–17]. Metaphylaxis is a proven technique in reducing morbidity and mortality among high-risk beef cattle, but its usage is highly variable when evaluated across populations and may influence the development of multidrug antimicrobial resistance [3, 17–19]. Therefore, the evaluation of gene expression and associated mechanisms of beef cattle at arrival may lead to the development of rapid diagnostics which accurately predict BRD development and severity. The development of a predictive test could allow for better assessment of BRD risk and reduce overall antimicrobial usage.

RNA sequencing (RNA-Seq) has become an invaluable tool for evaluated genomic and gene-by-environment associations related to clinical disease [20–22]. Previous studies have detected differentially expressed genes and enriched mechanisms associated with peak clinical disease after experimental challenge with known BRD-associated pathogens and related to naturally occurring disease [23–28]. Additionally, our previous research has defined genes and pathways related to pro-inflammatory mediation/resolution and antiviral defense to be increased at arrival in high-risk beef cattle that resist or succumb to naturally occurring BRD, respectively [29–31]. These studies have provided a better understanding of the host-disease complex relationship and created a defined record of target molecules involved in BRD, but their potential use as gene expression markers requires substantiation through extensive testing for classification accuracy and mechanistic delineation.

Candidate biomarker development remains a leading area of medical research, as technologies continue to improve, and the understanding of patient heterogeneity and dynamic changes associated with pathology are primary focuses of therapeutic advancement. Modern panels are developed in a multi-marker approach, as single-molecule diagnostics lack clinical significance and pathological representation [32]. Furthermore, single-molecule diagnostics often fail to define individuals within subgroups or cohorts of disease, as the intricacies of pathological severity and temporal association are frequently missed [32, 33]. Thus, we developed a multi-marker mRNA expression panel to evaluate previously identified differentially expressed genes and enriched mechanisms in high-risk post-weaned beef cattle at facility arrival. We hypothesized and corroborated that specific genes related to host immune and inflammatory responses are differentially expressed in equivocal manner to previous BRD host transcriptome studies. Here, we provide mRNA expressional information that represents BRD acquisition and severity across multiple populations of cattle, serving as a platform for the development of predictive diagnostics at arrival.

## Methods

### Animal use and enrollment

All animal use and procedures were approved by the Mississippi State University Animal Care and Use Committee (IACUC protocols #15–003 and #18–529) and carried out in accordance with relevant IACUC and agency guidelines and regulations. The information reported here is in accordance with Animal Research: Reporting of In Vivo Experiments (ARRIVE) guidelines (https://arriveguidelines.org). Whole blood samples were acquired from 397 cattle at facility arrival, spanning seven independent populations; five populations were cattle purchased from commercial livestock auctions within the state of Mississippi or neighboring states and housed at the H. H. Leveck Animal Research Center at Mississippi State University (Starkville, MS, USA; VD_15, *n* = 14; VD_17, *n* = 71; PS_19, *n* = 72; MH_19, *n* = 83; TA_20, *n* = 84) and two populations were cattle purchased from commercial livestock auctions within the state of Missouri or neighboring states and housed at the Professional Beef Services Research Facility in northeast Missouri (Canton, MO, USA; DG_17, *n* = 33; DG_18, *n* = 40). On day 0 for each population, blood samples were collected into RNA preservation tubes (Tempus Blood RNA Tubes, Thermo Fisher Scientific, Waltham, Massachusetts, USA) via jugular venipuncture and stored at − 80 °C until analysis. All cattle were given identification ear tags and confirmed to be negative for persistent infection with bovine viral diarrhea virus (BVDV) via ear notch antigen capture ELISA. Due to this study being performed in conjunction with seven independent experiments, metadata collection (average daily weight gain, pen assignment, vaccination/anthelminthic administration, etc.) varied by population.

Cattle within each population were assessed daily for clinical signs of BRD by trained staff. Cattle identified with clinical BRD were assigned a severity score of 0–4, adapted from the scoring system described by Holland and colleagues [34]. Necessary antimicrobial therapy for cattle housed in Mississippi was instituted as previously described [19]. Necessary antimicrobial therapy for cattle housed in Missouri followed a regimen of gamithromycin (Zactran; Boehringer-Ingelheim Animal Health USA, Duluth, GA, USA) and vitamin C, enrofloxacin (Baytril, Bayer Animal Health, Shawnee Mission, KS, USA) and vitamin C, or florfenicol with flunixin meglumine (Resflor Gold, Merck Animal Health, Madison, NJ, USA); all drugs were given at labeled dosage and route of administration. After the third antimicrobial treatment cattle with signs of predetermined endpoints indicative of unlikely recovery were evaluated by project veterinarians, and euthanized via methods approved by the American Veterinary Medical Association guidelines on euthanasia if necessary. All cattle that died or were euthanized were subjected to necropsy to identify grossly visible abnormalities of lungs and other organ systems. Clinical endpoints included severe dyspnea, dull mentation, and/or weakness leading to inability to take in feed and water adequate to sustain life.

### A priori and *post-hoc* power calculations

A priori and post hoc power analyses were performed with G*Power v3.1.9.7 [35] for sample size estimation to assess Healthy versus BRD gene expression and retrospective power estimation for the constructed decision tree model. Means and standard deviation observed between ΔCt values generated from RT-qPCR analysis of *ALOX15* [29] was utilized for a priori power calculation, accounting for an alpha of 0.05 and power (1-β error probability) of 0.95; power was calculated with a two-tailed t-Test of the mean differences between Healthy and BRD cattle. Two test post hoc analysis for the decision tree models utilized the proportions of correctly identified treated (BRD) and non-treated (Healthy) cattle.

### Sample selection and RNA processing

A total of 240 samples were selected for RNA isolation and gene expression analysis. One hundred sixteen samples from cattle never receiving treatment throughout their associated study (Healthy) were randomly selected across all seven populations with the RANDBETWEEN function in Excel 2016 (Microsoft Corp, Redmond, WA, USA). One hundred twenty-four samples from cattle treated at least once within 28 days of arrival (BRD) were selected and further stratified for severity based on treatment frequency. Of the 124 BRD samples, 33 samples from cattle treated twice or more and/or died (Treated_2+) were available and prioritized for analysis; the remaining 91 BRD samples were randomly selected with the RANDBETWEEN function in Excel 2016 from cattle treated once within 28 days of arrival and never treated again (Treated_1). Additional information regarding cattle selected for gene expression analysis is found in Supplementary Table S1.

RNA from samples collected into RNA preservation tubes (3 mL blood + 6 mL buffer) was isolated with Tempus Spin RNA Isolation kits (Thermo Fisher Scientific, Waltham, Massachusetts, USA) according to manufacturer’s instructions. RNA quantification, quality, and integrity was assessed with a NanoDrop ND-1000 Spectrophotometer (Thermo Fisher Scientific, Waltham, Massachusetts, USA) and Agilent 2100 Bioanalyzer system (Agilent Technologies, Santa Clara, CA, USA) at the Emory Integrated Genomics Core (EIGC; Emory University, Atlanta, GA, USA). RNA isolation integrity and concentration values for all samples are found in Supplementary Table S2. Samples having a concentration below 10 ng/μL were adjusted to > 10 ng/μL with a SpeedVac vacuum concentrator (Thermo Fisher Scientific, Waltham, Massachusetts, USA).

### Gene selection and NanoString nCounter assay

A custom NanoString nCounter (NanoString Technologies, Seattle, WA, USA) mRNA probe set was created to analyze the expression of 56 mRNA molecules of interest, and also four housekeeping genes (Supplementary Table S3). All genes selected were identified as differentially expressed in our previous studies and have known biological relevance related to immune, metabolic, and/or inflammatory systems [29–31]. Additionally, the panel included six positive and eight negative spike-in controls implemented by EIGC. Samples were prepared onto a 96-well plate and analyzed with the nCounter SPRINT Profiler (NanoString Technologies, Seattle, WA, USA), according to manufacturer’s protocol. Briefly, samples were hybridized to target-specific reporter and capture probes overnight at 65 °C in a thermocycler. Following hybridization, 30–35 μL of each hybridized product was pipetted to the nCounter cartridge, sealed, and loaded into the drawer of the SPRINT Profiler. A maximum field of view (FOV) sensitivity of 555 was selected for expressional capture.

### nCounter analysis

Reporter code counts (RCC) and reporter library file (RLF) obtained from the nCounter SPRINT Profiler were utilized for gene expression analysis though the nSolver Advanced Analysis Software v4.0 (NanoString Technologies, Seattle, WA, USA; https://www.nanostring.com/products/analysis-solutions/ncounter-analysis-solutions/). Background correction, quality assessment, and inter-sample normalization was performed in accordance with the Gene Expression Data Analysis Guidelines (MAN-C0011–04). Imaging quality control measurements were set to flag any lane failing to have a registered FOV above 75%. Binding density was flagged if any lane recorded an optical feature per square micron outside the range of 0.1 to 1.8. Positive control linearity, used to assess the efficiency of hybridization, was flagged if any resulting count was below a correlation of 0.95. Positive control limit of detection flagged any sample if the 0.5 fM positive control counts were ≤ 2 SD above the mean of the negative controls. Background thresholds were set to the mean of the manufactured negative controls plus two standard deviations. Positive control normalization was performed using the geometric mean to compute normalization factors for each sample; lanes with a normalization factor outside of the 0.3–3.0 range were flagged. Codeset content normalization was performed with the geometric mean of the four housekeeping genes, flagging lanes if the normalization factor was outside of the 0.1–10.0 range. In total, six samples (72_VD_15, 118_VD_17, 147_VD_17, 188_VD_17, 143_PS_19, and 205_TA_20) were removed from further analysis due to low quality. Normalized mRNA counts were analyzed for differential expression in four comparative analyses: Healthy versus BRD, Treated_1 versus Healthy, Treated_2+ versus Healthy, and Treated_2+ versus Treated_1. Differential expression analysis was performed with the normalized count data via the nSolver Advanced Analysis module, accounting for study (VD_15, VD_17, etc.) as a potential confounding variable; study-level effect was incorporated into the data analysis model to ensure it did not skew the parameter estimation for differential expression analysis and reduce the likelihood of type I error. Information regarding confounding variables and estimation of model coefficients are found in the nCounter Advanced Analysis User Manual, pp. 46–52 (MAN-10030-03; https://www.nanostring.com/wp-content/uploads/2020/12/MAN-10030-03_nCounter_Advanced_Analysis_2.0_User_Manual.pdf). Differentially expressed genes (DEGs) were determined to be significant with a *p*-value of < 0.05. Gene expression data produced in this study were submitted to the National Center for Biotechnology Information Gene Expression Omnibus (NCBI-GEO) under accession GSE179536.

### Statistical analyses and data characterization

Due to discrepancies in metadata recording across all seven studies, average daily gain in pounds at the end of each study, when cattle were sold at study conclusion (ADGend), was assessed for distributive differences associated with frequency of treatment. Residual normality and assumption of equal variance was assessed in R v4.0.4 with the Shapiro-Wilk and Bartlett’s test, respectively. When accounting for disease severity, the residuals were found to be non-normally distributed. A one-way Kruskal-Wallis test was used to test the relationship between ADGend and severity of disease, blocking for study as a potential confounding variable. Pairwise comparisons were assessed, accounting for familywise error rate with the Bonferroni correction method; any comparison with an adjusted *p*-value of < 0.05 was considered significant.

Normalized gene expression values were exported from nSolver as a comma separated values file. Counts were imported into R v4.0.4 and converted to log2 count-per-million (log2CPM) values for downstream statistical and classificational analyses. Heatmap visualization and unsupervised hierarchical clustering of z-score-scaled log2CPM gene expression values were performed with the Bioconductor package pheatmap v1.0.12 [36], utilizing Ward’s minimum variance method of unsupervised hierarchical clustering on Minkowski distances and Pearson correlation coefficients for samples and DEGs, respectively. Multi-level modeling (MLwiN v2.25, Centre for Multilevel Modelling, University of Bristol, Bristol, EN, UK) was used to assess the proportion of variance at each level of disease status (BRD, Healthy) within study and severity of disease (Healthy, Treated_1, Treated_2+) within study. Variance partition coefficients were calculated to observe the proportion of the response variation that lay within each level of the hierarchy by dividing the variation within a level by the sum of all the variation within the model. Equal variance between genes by disease status was assessed using the Levene’s test for homogeneity of variance in the general linear model procedure of SAS v9.2 (SAS Institute, Cary, NC, USA), in order to evaluate for outlier-driven gene expression. An a priori level of significance was set at an alpha of 0.10. Visual assessment of the conditional residuals confirmed the assumption of normal distribution. Remaining data visualization was conducted with ggplot2 v3.3.3 [37] and UpSetR v1.4.0 [38, 39]. Graphical color scaling was performed with viridis v0.6.1 [40] to allow ease of visual interpretation for individuals with color blindness.

Counts from uniquely identified DEGs were utilized for multiclass receiver operating characteristic (ROC) curve analysis, recording aggregate areas under the curve (AUC) and cutoff values used to distinguish disease severity cohorts. ROC curve analyses were conducted with the Bioconductor package pROC v1.17.0 [41]. Classification between the three groups (Healthy, Treated_1, and Treated_2+) was evaluated as “excellent” (AUC: > 0.900), “good” (AUC: 0.899–0.800), “fair” (AUC: 0.799–0.700), “poor” (AUC: 0.699–0.600), or “failed” (AUC: < 0.600). An empirical decision tree model was constructed to identify the maximum predictive accuracy of disease severity classification from DEGs, utilizing log2CPM value thresholds generated from the ROC analysis of Treated_2+ versus Healthy cattle. Correctly identified and misclassified Healthy and Treated_1 cattle from the resulting decision tree model were assessed for differences in ADGend values via two-tailed Student’s t-Test with an a priori level of significance set to an alpha of 0.10.

## Results

**Fig. 1:**
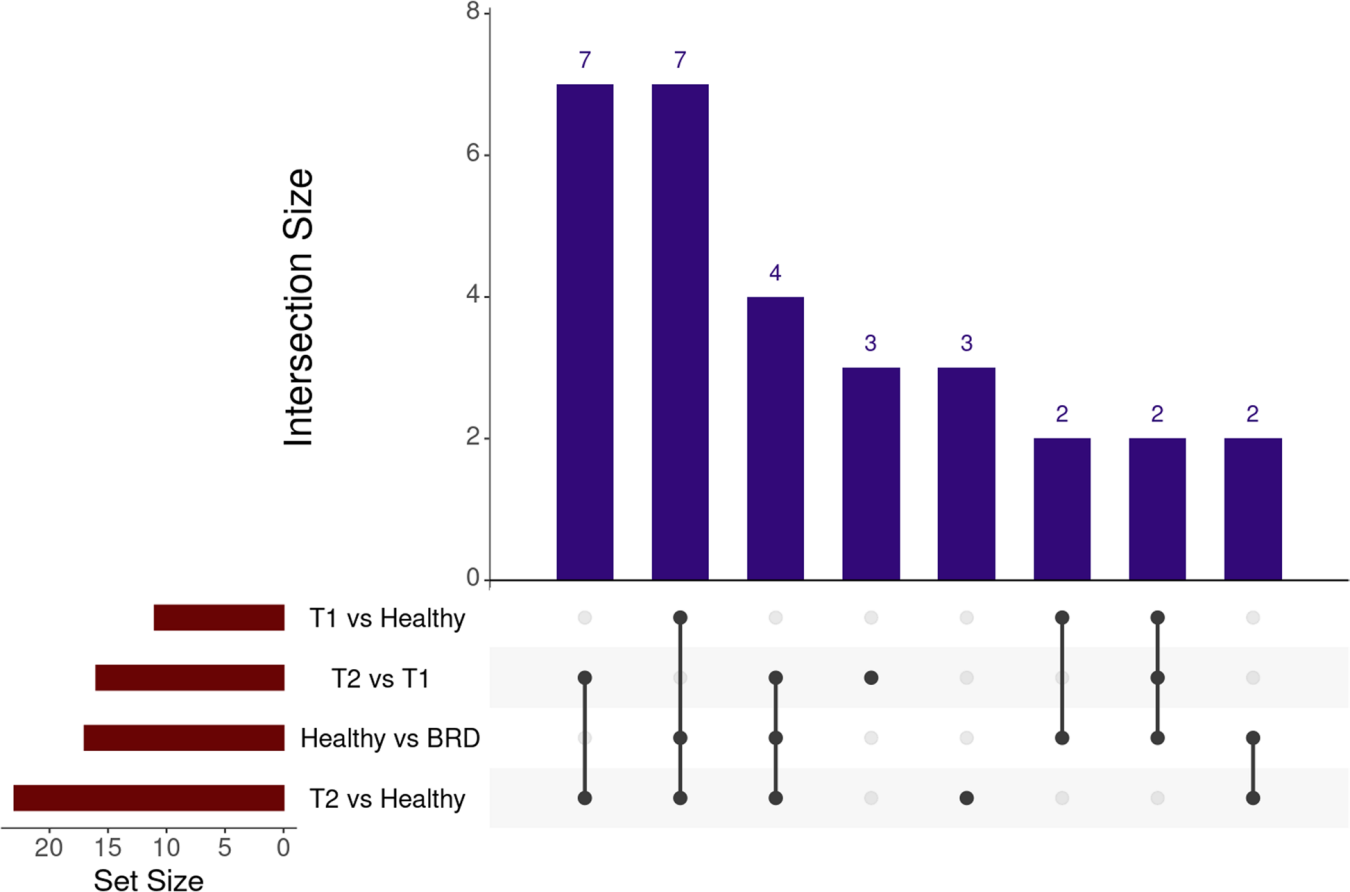
Overlap of the 30 unique DEGs identified from nSolver analysis between the four comparative analyses. Interaction size represents the quantity of overlapping genes within and between each analysis. Set size represents the total number of DEGs identified in each analysis. Healthy are cattle never diagnosed nor received antimicrobial therapy for clinical BRD. T1 are cattle treated for clinical BRD once within 28 days of arrival. T2 are cattle having been treated at least two times within 28 days of arrival for and/or died following a diagnosis of BRD. BRD are T1 and T2 cattle combined into one group

A priori power analysis concluded that 210 samples would be required (Healthy, *n* = 105; BRD, *n* = 105) to achieve a power level of 0.95. After removal of samples that failed quality control assessment, 115 Healthy and 119 BRD (Treated_1, *n* = 89; Treated_2+, *n* = 30) samples remained for differential expression analysis. Seventeen genes were identified as differentially expressed between Healthy and BRD cattle (Supplementary Table S4). Healthy cattle, when compared to BRD, possessed increased expression of genes associated with anti-inflammatory processes (*CPB2* and *IL5RA*), specialized pro-resolving mediator (SPM) metabolism (*ALOX15* and *HPGD*), lymphocytic maturation (*LOC100297044*, *GCSAML*, and *KLF17*), and antimicrobial peptide production (*CATHL3*, *GZMB*, and *LTF*); BRD cattle possessed increased expression of genes associated with type I interferon production (*HERC6* and *IFI6*), alternative complement (*CFB*), and granulocyte adhesion/cell-cell interaction (*DSG1*, *LRG1*, and *MCF2L*). Pairwise analysis of each severity cohort revealed 11, 22, and 16 genes to be differentially expressed between Treated_1 versus Healthy, Treated_2+ versus Healthy, and Treated_2+ versus Treated_1 comparisons, respectively (Supplementary Tables S5, S6, S7). *CFB* was decreased in expression in Healthy cattle when independently compared to both Treated_1 and Treated_2+ cattle. Type I interferon-associated genes (*HERC6*, *IFI6*, *ISG15*, and *MX1*) were increased in Treated_2+ cattle compared to Healthy, but only *HERC6* was considered differentially expressed between Treated_2+ and Treated_1 cattle. *ALOX15* and *HPGD* were decreased in Treated_1 cattle compared to Healthy, but were not considered differentially expressed between Treated_2+ and Healthy cattle. In total, 30 genes were uniquely identified as differentially expressed in at least one comparative analysis (Fig. 1).

**Fig. 2:**
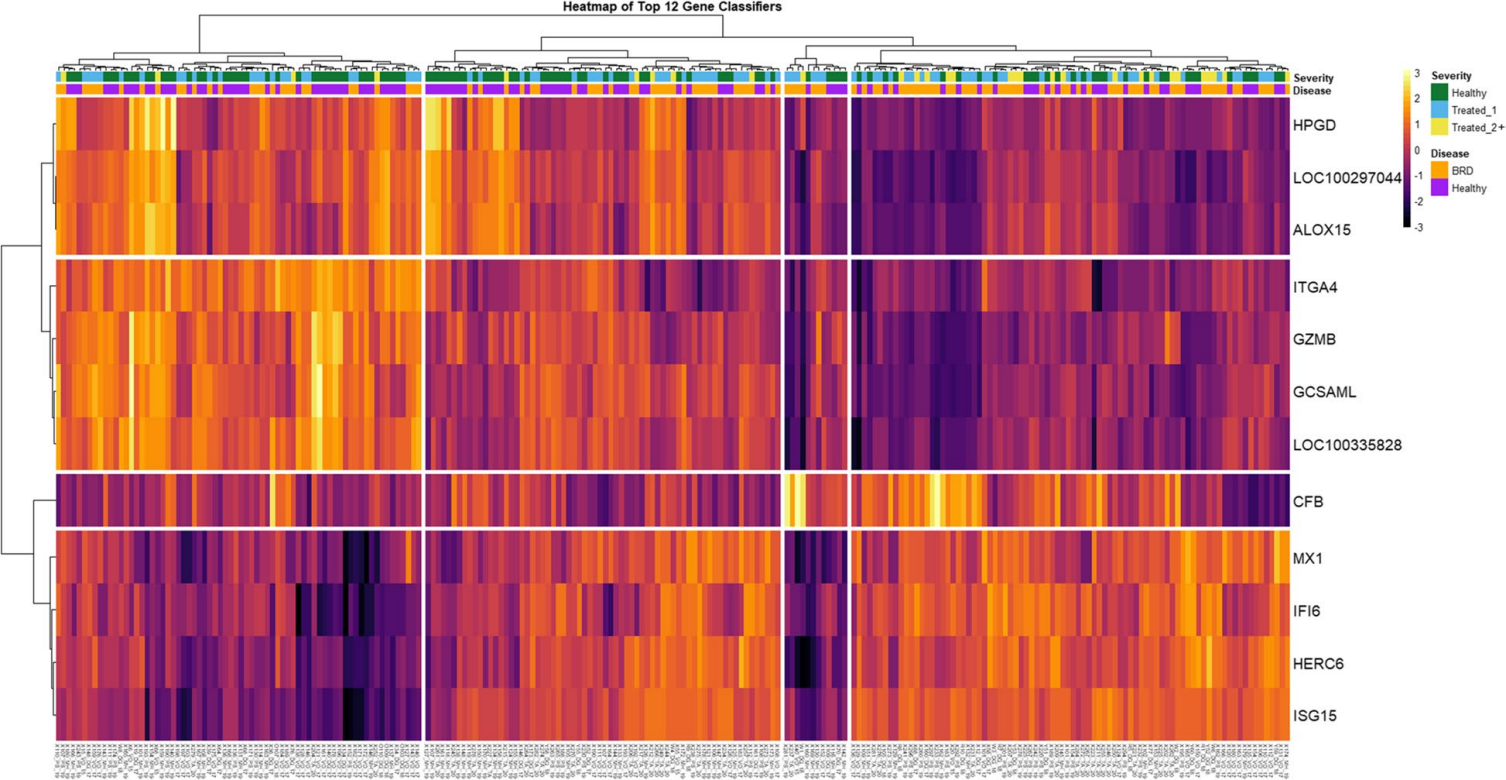
Heatmap and unsupervised hierarchical clustering of the gene expression from twelve select DEGs. Expression values (− 3 to + 3) were calculated from z-score calculation of nCounter normalized expression values for each gene. Samples were further labeled for disease status (BRD, Healthy) and severity (Healthy, Treated_1, Treated_2+). Yellow/white: relative high expression; purple/black: relative low expression

A heatmap was generated for twelve DEGs that best stratified disease severity, based on hierarchical clustering of gene expression patterns across samples (Fig. 2). Clustering of Pearson correlation coefficients was used to stratify the twelve genes into four expressional arrays, which consisted of type I interferon-associated genes (*HERC6*, *IFI6*, *ISG15*, and *MX1*), complement factor B (*CFB*), SPM and leukocyte-associated genes (*ALOX15*, *HPGD*, and *LOC100297044*), and cell adhesion/lymphocyte-associated genes (*ITGA4*, *GZMB*, *GCSAML*, and *LOC100335828*). BRD cattle, specifically within the Treated_2+ classification, appeared to cluster more to the right side of the heatmap associated with higher levels of type I interferon-associated gene expression and lower levels of SPM and cell adhesion/lymphocyte-associated gene expression. However, Healthy and Treated_1 cattle appeared more similar in expressional patterns compared to Treated_2+ cattle.

**Fig. 3:**
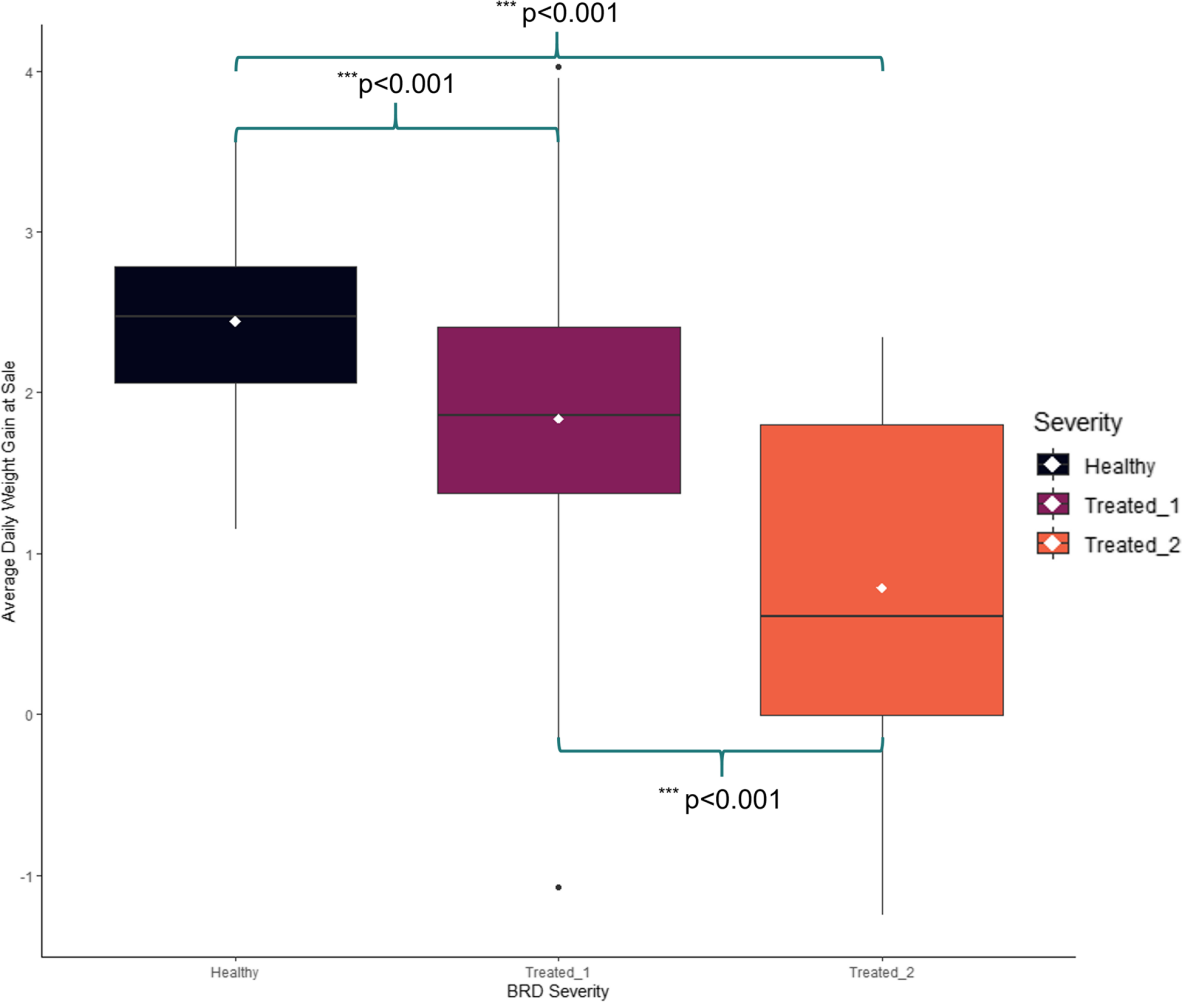
Distributive differences in average daily weight gain in pounds at time of sale (ADGend). Boxplots limits are associated with the first (lower) and third (upper) quartiles. Horizontal lines within the boxplots represent the ADGend median for each cohort. White points within the boxplots represent ADGend mean for each cohort. Whiskers for each boxplot extend to 1.5 times the interquartile ranges. Any black point outside the vertical range of whiskers represents outlier individuals within the associated cohort

ADGend values were used to assess growth performance across the three disease severity cohorts. One-way Kruskal-Wallis testing revealed a significant difference in the distribution of average daily weight gain across the three cohorts (F = 24.699, *p* < 0.001). Pairwise comparisons of the three groups demonstrated an overall decrease in weight gain with increased frequency of treatment (*p* < 0.001; Fig. 3).

**Fig. 4:**
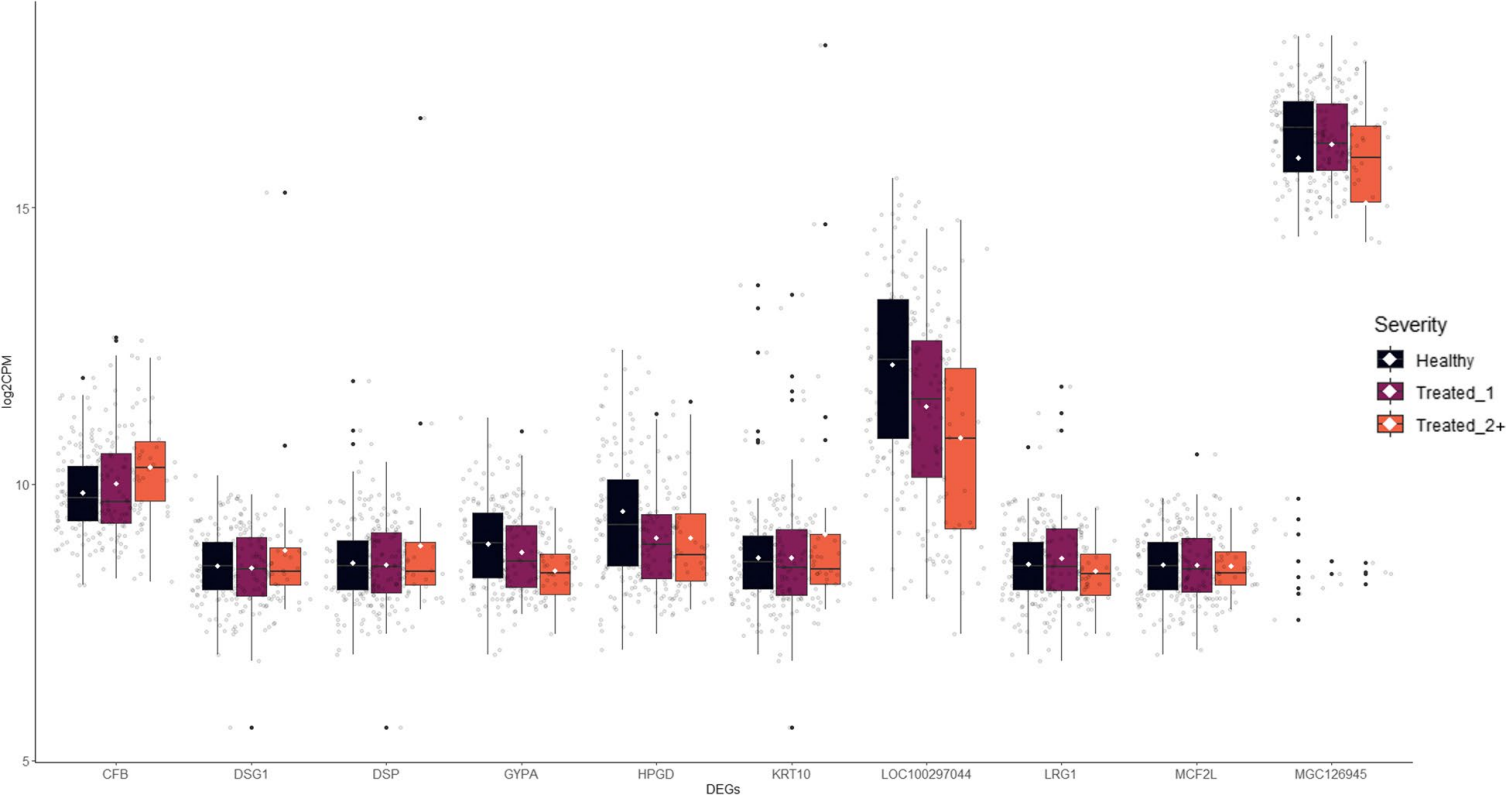
Boxplots of the log2CPM gene expression for the ten genes with unequal variance across severity. Genes were identified through the Levene’s test for homogeneity of variance (*p* < 0.10). Boxplots limits are associated with the first (lower) and third (upper) quartiles. Horizontal lines within the boxplots represent the median log2CPM for each cohort. White points within the boxplots represent log2CPM mean for each cohort. Whiskers for each boxplot extend to 1.5 times the interquartile ranges. Any black point outside the vertical range of whiskers represents outlier individuals within the associated cohort

Multi-level modeling identified the proportion of variance was highest when factoring for disease severity, with an average of 92.50% lying between disease severity cohorts within study (Supplementary Table S8). *IFI6* possessed the highest discrepancy in variance, as 16.97% lay between studies and 83.03% lay between disease cohorts within study. *MGC126945* possessed the highest level of variance between disease cohorts within study (100%). Analysis for homogeneity of variance across severity of disease revealed that unequal variance existed for ten DEGs (*p* < 0.10): *CFB*, *DSG1*, *DSP*, *GYPA*, *HPGD*, *KRT10*, *LOC100297044*, *LRG1*, *MCF2L*, and *MGC126945* (Fig. 4). *CFB*, *GYPA*, *LOC100297044*, *LRG1*, and *MCF2L* demonstrated more homogeneous gene expression within the Treated_2+ cohort compared Healthy and Treated_1 cattle. Notably, *DSG1*, *DSP*, *HPGD*, *KRT10*, and *MGC126945* were more variable in terms of gene expression within and between disease cohorts. Complete results for homogeneity of variance analysis across severity cohorts is found in Supplemental Fig. S1 and Supplemental Table S8.

All 30 unique DEGs were selected for multiclass ROC curve evaluation to determine log2CPM cutoffs and discriminative capability for predicting BRD severity outcomes (Table 1). For univariate ROC analysis, all but three DEGs (*DSG1*, *DSP*, and *KRT10*) best discriminated Treated_2+ versus Healthy cattle, as opposed to Treated_1 versus Healthy or Treated_2+ versus Treated_1. Consequently, these three genes were shown to possess high inter-group variation and outlier-driven means associated with Treated_2+ (Fig. 4; mean versus median values). Eleven independently evaluated DEGs failed to discriminate Treated_2+ and Healthy cattle (AUC < 0.600). *LOC100297044* demonstrated good discrimination of Treated_2+ and Healthy cattle and was subsequently the top independent classifier (AUC: 0.868). To assess pathway-based expressional classification, two multivariable classifier models were constructed: type I interferon-associated (IFN) with *HERC6*, *IFI6*, *ISG15*, and *MX1* and SPM-associated with *ALOX15* and *HPGD*. IFN demonstrated good discrimination of Treated_2+ from Healthy (AUC: 0.730). SPM demonstrated poor discrimination of Treated_2+ from Healthy (AUC: 0.646). Multiclass ROC curve analyses by population for DEGs previously evaluated [31] and/or selected for decision tree construction are found in Supplemental Table S9.

**Table 1:** Receiver operator characteristic (ROC) curve analysis with area under the curve (AUC), sensitivity, and specificity

Comparison	Gene	AUC	Cutoff	Sensitivity	Specificity
Treated_1 vs Healthy	ALDH1A1	0.520	8.115	0.360	0.730
Treated_2+ vs Healthy		0.533	8.585	0.667	0.478
Treated_2+ vs Treated_1		0.494	8.143	0.767	0.371
Treated_1 vs Healthy	ALOX15	0.590	12.855	0.966	0.191
Treated_2+ vs Healthy		0.635	8.703	0.400	0.904
Treated_2+ vs Treated_1		0.554	8.415	0.333	0.865
Treated_1 vs Healthy	BGN	0.514	8.115	0.348	0.730
Treated_2+ vs Healthy		0.527	8.585	0.667	0.478
Treated_2+ vs Treated_1		0.508	8.503	0.633	0.472
Treated_1 vs Healthy	CATHL3	0.540	8.388	0.416	0.696
Treated_2+ vs Healthy		0.625	8.585	0.633	0.626
Treated_2+ vs Treated_1		0.577	8.878	0.767	0.436
Treated_1 vs Healthy	CD27	0.524	13.946	0.382	0.730
Treated_2+ vs Healthy		0.659	14.306	0.800	0.557
Treated_2+ vs Treated_1		0.613	14.310	0.800	0.506
Treated_1 vs Healthy	CFB	0.483	9.629	0.483	0.583
Treated_2+ vs Healthy		0.656	10.058	0.667	0.661
Treated_2+ vs Treated_1		0.618	9.673	0.767	0.494
Treated_1 vs Healthy	CPB2	0.588	8.714	0.562	0.652
Treated_2+ vs Healthy		0.679	8.793	0.767	0.635
Treated_2+ vs Treated_1		0.573	9.020	0.833	0.404
Treated_1 vs Healthy	DSG1	0.516	8.115	0.360	0.739
Treated_2+ vs Healthy		0.499	8.792	0.733	0.374
Treated_2+ vs Treated_1		0.475	0.900	0.800	0.281
Treated_1 vs Healthy	DSP	0.499	8.115	0.337	0.739
Treated_2+ vs Healthy		0.494	8.585	0.600	0.487
Treated_2+ vs Treated_1		0.489	8.482	0.567	0.517
Treated_1 vs Healthy	GCSAML	0.543	9.225	0.337	0.774
Treated_2+ vs Healthy		0.750	9.340	0.700	0.739
Treated_2+ vs Treated_1		0.694	9.586	0.800	0.584
Treated_1 vs Healthy	GYPA	0.571	8.800	0.584	0.565
Treated_2+ vs Healthy		0.692	8.801	0.800	0.565
Treated_2+ vs Treated_1		0.612	8.881	0.833	0.404
Treated_1 vs Healthy	GZMB	0.609	10.318	0.618	0.583
Treated_2+ vs Healthy		0.732	9.786	0.700	0.696
Treated_2+ vs Treated_1		0.640	9.488	0.633	0.652
Treated_1 vs Healthy	HERC6	0.575	14.942	0.663	0.504
Treated_2+ vs Healthy		0.741	15.352	0.600	0.835
Treated_2+ vs Treated_1		0.660	15.352	0.600	0.719
Treated_1 vs Healthy	HPDG	0.608	9.582	0.809	0.391
Treated_2+ vs Healthy		0.617	8.964	0.700	0.609
Treated_2+ vs Treated_1		0.522	8.952	0.700	0.494
Treated_1 vs Healthy	IFI6	0.585	15.478	0.494	0.687
Treated_2+ vs Healthy		0.699	15.496	0.733	0.687
Treated_2+ vs Treated_1		0.631	15.630	0.667	0.640
Treated_1 vs Healthy	IL5RA	0.622	10.898	0.775	0.443
Treated_2+ vs Healthy		0.651	8.907	0.433	0.852
Treated_2+ vs Treated_1		0.555	8.921	0.433	0.742
Treated_1 vs Healthy	ISG15	0.545	16.689	0.708	0.417
Treated_2+ vs Healthy		0.699	17.930	0.600	0.800
Treated_2+ vs Treated_1		0.657	17.606	0.733	0.596
Treated_1 vs Healthy	ITGA4	0.540	15.461	0.303	0.817
Treated_2+ vs Healthy		0.676	15.674	0.633	0.713
Treated_2+ vs Treated_1		0.622	15.921	0.733	0.551
Treated_1 vs Healthy	KLF17	0.530	8.115	0.337	0.791
Treated_2+ vs Healthy		0.596	8.589	0.700	0.522
Treated_2+ vs Treated_1		0.552	8.996	0.867	0.303
Treated_1 vs Healthy	KRT10	0.509	8.115	0.337	0.748
Treated_2+ vs Healthy		0.471	8.444	0.500	0.557
Treated_2+ vs Treated_1		0.460	9.283	0.833	0.225
Treated_1 vs Healthy	LOC100297044	0.629	13.045	0.899	0.313
Treated_2+ vs Healthy		0.868	9.952	0.467	0.904
Treated_2+ vs Treated_1		0.577	9.981	0.467	0.764
Treated_1 vs Healthy	LOC100335828	0.529	9.695	0.371	0.730
Treated_2+ vs Healthy		0.731	9.784	0.700	0.704
Treated_2+ vs Treated_1		0.688	10.079	0.800	0.562
Treated_1 vs Healthy	LRG1	0.479	8.116	0.315	0.739
Treated_2+ vs Healthy		0.566	8.585	0.700	0.487
Treated_2+ vs Treated_1		0.579	8.996	0.867	0.337
Treated_1 vs Healthy	LTF	0.613	9.191	0.629	0.635
Treated_2+ vs Healthy		0.727	8.885	0.667	0.551
Treated_2+ vs Treated_1		0.615	9.211	0.800	0.617
Treated_1 vs Healthy	MCF2L	0.510	8.300	0.427	0.635
Treated_2+ vs Healthy		0.532	8.585	0.667	0.487
Treated_2+ vs Treated_1		0.514	8.996	0.867	0.292
Treated_1 vs Healthy	MGC126945	0.532	16.117	0.506	0.643
Treated_2+ vs Healthy		0.638	16.293	0.700	0.591
Treated_2+ vs Treated_1		0.617	15.081	0.267	0.933
Treated_1 vs Healthy	MS4A2	0.506	8.115	0.326	0.783
Treated_2+ vs Healthy		0.608	8.591	0.700	0.548
Treated_2+ vs Treated_1		0.591	8.997	0.867	0.404
Treated_1 vs Healthy	MX1	0.581	17.544	0.427	0.800
Treated_2+ vs Healthy		0.677	17.420	0.667	0.713
Treated_2+ vs Treated_1		0.598	17.643	0.500	0.742
Treated_1 vs Healthy	SLC18A2	0.494	8.998	0.281	0.783
Treated_2+ vs Healthy		0.563	8.585	0.700	0.478
Treated_2+ vs Treated_1		0.552	8.503	0.667	0.494
Treated_1 vs Healthy	SPP1	0.514	8.115	0.348	0.730
Treated_2+ vs Healthy		0.529	8.585	0.667	0.487
Treated_2+ vs Treated_1		0.508	8.996	0.833	0.281
Treated_1 vs Healthy	IFN (HERC6, IFI6, ISG15, MX1)	0.578	64.739	0.652	0.539
Treated_2+ vs Healthy		0.730	66.093	0.733	0.748
Treated_2+ vs Treated_1		0.661	65.926	0.767	0.618
Treated_1 vs Healthy	SPM (ALOX15, HPGD)	0.598	17.288	0.292	0.887
Treated_2+ vs Healthy		0.646	17.407	0.433	0.878
Treated_2+ vs Treated_1		0.548	17.787	0.467	0.685

ROC evaluation was used to generate log2CPM cutoffs for decision tree construction and assess DEGs as single-molecule predictors of BRD severity

**Fig. 5:**
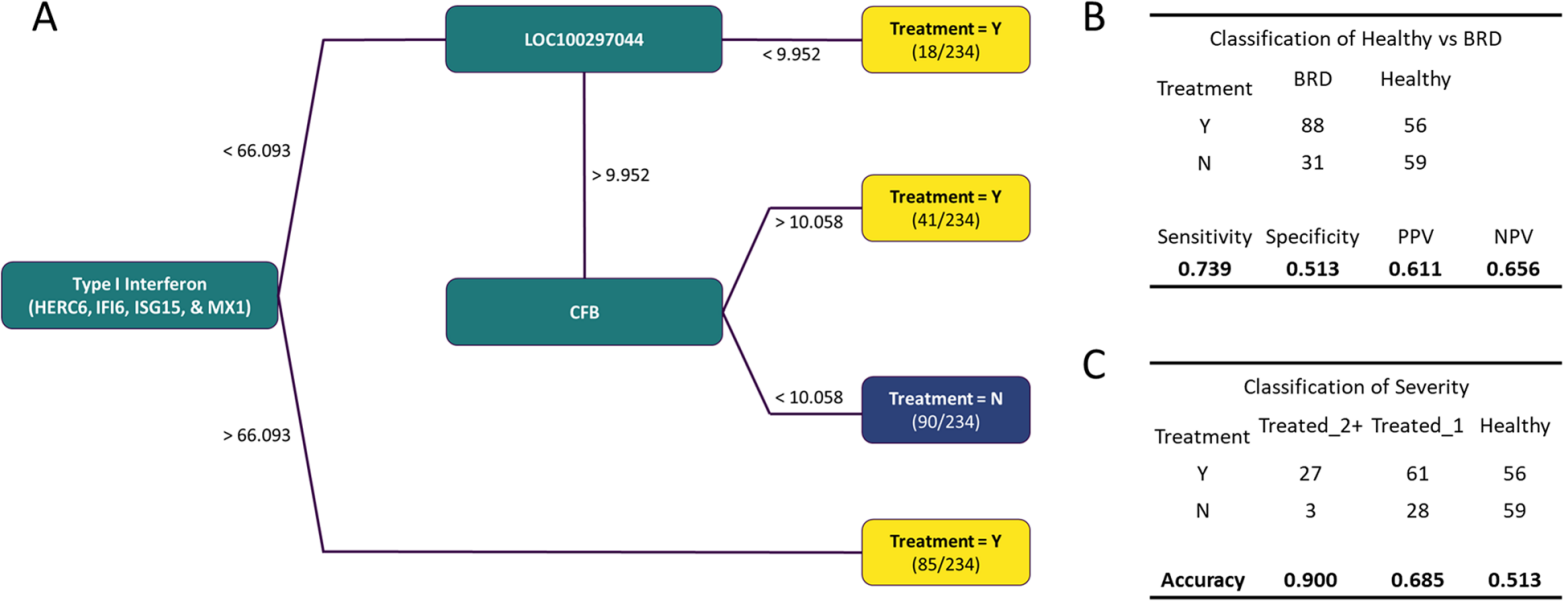
Gene expression decision tree model constructed from log2CPM cutoffs derived from ROC curve evaluation. **A** Combined type I interferon genes (*HERC6, IFI6, ISG15,* and *MX1*) serve as the root of the decision tree, with an additive log2CPM cutoff of 66.093. Individuals with a value above the threshold are marked as needing BRD treatment, and below the threshold move to the *LOC100297044* leaf. Those within the *LOC100297044* leaf below a threshold of 9.952 are marked as needing BRD treatment, and above the threshold move to the *CFB* leaf. Those within the *CFB* leaf above a threshold of 10.058 are marked as needing BRD treatment, and those below the threshold are considered Healthy. **B** Diagnostic accuracy table for the assessment of identified Healthy and BRD individuals. **C** Diagnostic accuracy table for the assessment of identified Treated_2+, Treated_1, and Healthy individuals

An at-arrival treatment decision tree model was constructed from these DEG classifiers, utilizing log2CPM value expression levels for all individuals (Fig. 5A). *Post-hoc* power analysis of the resulting decision tree indicated a power of 0.94 for determining Healthy and BRD cattle. Overall, the treatment model successfully identified 73.9% (88/119) of all BRD cattle and both predictive values, dependent upon the distribution of Healthy and BRD within this study, were above 61.0% (Fig. 5B). Notably, when stratifying for disease severity, the model accurately identified Treated_2+ and Treated 1 individuals with 90.0 and 68.5% accuracy, respectively (Fig. 5C). Statistical assessment of the Healthy cattle misclassified as needing treatment (*n* = 56) compared to correctly identified Healthy cattle (*n* = 59) revealed no difference in ADGend (*p* = 0.193). However, Treated_1 cattle misclassified by the decision tree as not needing treatment (i.e., Healthy) possessed a significantly higher ADGend (*n* = 28; x̅=2.091, σ_x_ = 0.844) than those correctly identified (*n* = 61; x̅=1.728, σ_x_ = 0.850) (*p* = 0.084).

## Discussion

Trained individuals within conventional beef production systems handle cattle at a limited number of time points, predominately at arrival, to reduce animal stress and minimize labor demand. Coupled with the complex nature of BRD and lack of a gold standard diagnostic test, these systems have difficulty determining likelihood of disease acquisition and severity on an intra-population scale. Accordingly, our previous RNA-Seq studies were conducted to better characterize the at-arrival host response in cattle related to subsequent development and treatment of naturally-acquired BRD within the first 28 days of facility placement [29–31]. RNA-Seq studies employ robust statistical and mathematical models in an effort to identify potential molecular targets and mechanisms that may improve disease understanding, detection, and therapy [42, 43]. Such studies reduce the overall dimensionality of host expression and allow for the determination of a finite number of molecules to assess in future studies. However, the development of disease prediction or prognostic models requires rigorous testing and can be resource intensive, as gene-by-environment interactions and disease temporality are difficult to account for in developing predictive models [33, 44]. Therefore, we focused on corroborating differential expression and retrospectively classifying the predictive capability of mRNA molecules previously identified by RNA-Seq, but through a method feasible for use in large numbers of cattle. To this purpose, we utilized NanoString nCounter mRNA profiling as a less variable and higher throughput method of evaluating specific gene expression, compared to the commonly utilized alternative, RT-qPCR [45–49]. To our knowledge, this study is the first to substantiate selective at-arrival gene expressional patterns previously associated with BRD acquisition and severity, utilizing blood samples from beef cattle across multiple independent populations.

A limitation of this work is that we aimed to identify at-arrival gene expression profiles that predicted future treatment for BRD based on clinical assessment, which can lack diagnostic sensitivity [13, 14]. While we recognize that the visual identification of clinical BRD is relatively insensitive and potentially confounded by study location and associated experimental conditions, our statistical analyses and modeling were conducted to account for this effect. Furthermore, analysis of weight gain records at time of sale (ADGend) demonstrated an objective loss in production that was associated with increased frequency of treatment, in agreement with previous research (Fig. 3) [50, 51], confirming the significance of the BRD diagnoses in this study.

In total, 30 unique genes were determined to be differentially expressed across all comparisons (Fig. 1; Supplemental Tables S4, S5, S6, S7). Cattle never diagnosed with clinical BRD possessed increased expression of genes broadly associated with leukocyte/granulocyte development and differentiation (*GCSAML*, *IL5RA*, *KLF17*, *LOC100297044* (CCL14), and *LOC100335828* (CD200R1)), SPMs (*ALOX15* and *HPGD*), anti-inflammatory/apoptotic processes (*CPB2* and *ITGA4*), and antimicrobial peptides (*CATHL3*, *GZMB*, and *LTF*). *CD200R1*, *CCL14*, *GCSAML*, and *IL5RA* have previously been identified as chemokines for macrophage activity and enhanced lymphocyte migration and survival [52–56]. SPMs, while not widely explored in ungulates, are categorized into two primary categories based on the metabolism of arachidonic acid (lipoxins) or essential polyunsaturated fatty acids (resolvins, maresins, and protectins) [57, 58]. In other mammalian species, SPMs are involved in quelling prolonged pro-inflammatory events and promoting cellular/molecular homeostasis in the lower airways; these molecules are currently under investigation as therapeutic modalities in humans with sepsis or viral-induced pneumonia/acute respiratory distress syndrome (ARDS) [59–62]. *CPB2* and *ITGA4* are key regulators of pro-inflammation, as they are involved in modulating the complement system, degrading induced plasma anaphylatoxins, and reducing nitric oxide accumulation [63–68]. Cattle are capable of producing a myriad of well conserved innate peptides within the cathelicidin and defensin peptide families, such as *CATHL3*, *GZMB*, and *LTF*, which have oxygen-independent killing capacity against both Gram-positive and Gram-negative pathogens [69, 70]. These amphophilic molecules align specifically to bacterial cell walls via electrostatic interactions and penetrate into the cytoplasmic space, leading to DNA replication disruption and/or bacterial autolysis [71–74]. Collectively, these findings indicate that cattle that remain clinically healthy possessed enhanced immunological, anti-inflammatory, and microbial killing mechanisms at arrival, compared to cattle that require antimicrobial therapy within the first 28 days of arrival.

Cattle that would go on to develop BRD possessed increase expression of genes at arrival broadly associated with pro-inflammatory, granulocytic processes (*LRG1*, *MCF2L*, *SPP1*), alternative complement (*CFB*), and type I interferon signaling (*HERC6*, *IFI6*, *ISG15*, and *MX1*), when compared to healthy cattle. Notably, Treated_1 cattle only possessed increased expression of *CFB* and *LRG1* when compared to Healthy cattle. Therefore, these aforementioned genes and their associations are predominantly detected within Treated_2+ cattle. *LRG1* encodes for a leucine-rich glycoprotein specifically stored and secreted by neutrophils, which appears to be marker of early differentiation [75, 76]. *MCF2L* explicitly binds with RAC subfamily of Rho GTPases and may be critical in neutrophilic signal transduction [77, 78]. *SPP1* is a major secreted component of pro-inflammatory leukocytes and enhances cellular infiltration and fibrosis of the airways [79–81]. *CFB*, increased in both Treated_1 and Treated_2+ cattle when independently compared to Healthy cattle, encodes for the proenzyme of alternative complement. In human sera, *CFB* has been indicated in early, severe infectious disease, such as that induced by severe acute respiratory syndrome coronavirus 2 (SARS-CoV-2) [82, 83]. Additionally, *CFB* production is exaggerated in human airway epithelial cells following experimental asthma and rhinovirus challenge [84]. *HERC6*, *IFI6*, *ISG15*, and *MX1* encode for proteins induced by type I interferons, such as IFN-α, IFN-β, IFN-ω, and are typically produced by host cells in response to viral infection [85–87]. Cattle experimentally infected with BRD-associated viruses, such as bovine respiratory syncytial virus (BRSV) and bovine herpesvirus 1 (BHV-1), demonstrate increased expression of these genes during peak clinical presentation, when compared to sham controls [23–26]. While this appears to be a natural host (bovine) response to viral infection, our previous and current findings, coupled with the work of Sun and colleagues, indicate that anti-viral responses are indicative of early stage and, often, severe naturally-acquired BRD [27, 30]. Previous work indicated strong elevation and co-expression of these genes at arrival in cattle that died of BRD, with predicted interactions with pro-inflammatory cytokine production such as interleukin-6 and tumor necrosis factor-α [30, 88]. Taken together, our findings suggest that cattle that required multiple antimicrobial therapies, and/or died of naturally-acquired BRD, entered the production system with increased anti-viral defense mechanisms without the benefit of activation/elevation of mechanisms for pro-inflammatory regulation or resolution. However, further studies pairing host gene expression with the microbiome and/or virome of cattle at arrival are necessary, as the relationship and influence of microbial populations on host response as it relates to severe BRD (Treated_2+) are unknown.

To further clarify the relationship between DEGs and disease status, we employed hierarchical clustering and visualization with a gene expression heatmap (Fig. 2). Notably, Healthy and Treated_1 cattle were relatively indistinguishable based on the expression patterns of those 12 DEGs, but Treated_2+ cattle tended to cluster with the association of elevated type I interferon-associated gene expression and decreased SPM and lymphocyte proliferation-associated gene expression (Fig. 2, right side). This finding is similar to results found in our previous multipopulational transcriptome analysis, suggesting that Treated_2+ cattle are more distinct in terms of at-arrival gene expression identification compared to Healthy and Treated_1 cattle [31]. This finding was made more apparent when assessing multilevel modeling and inter-cohort variance, as Treated_2+ cattle tended to be more similar in gene expression compared to Healthy and Treated_1 cohorts, regardless of study-level effect (Figs. 4 and S1). *CFB*, a gene identified with unequal variance and representing both BRD acquisition and severity, was relatively homogeneous in terms of gene expression within Treated_2+ cattle, but disproportionate within the Healthy and Treated_1 cohorts. Outliers within the Healthy and Treated_1 cohorts tended to shift mean gene expression higher than the median and drove differences in variance observed between the three groups. The observed results of *CFB* may suggest that some cattle within the Healthy and Treated_1 cohort had subclinical BRD. Further evaluation of variance determined that *DSG1*, *DSP*, *KRT10*, and *MGC126945* appeared to be outlier-driven, implying unproportionate differential gene expression across populations, and ruling them out as candidate predictive classifiers for BRD.

Multiclass ROC curve analysis was utilized to generate cutoffs and assess classificational capability for each unique DEG; however, this demonstrated that a single-molecule classification model was not sufficient to capture the dynamic expressional patterns observed across all seven populations (Table 1). Notably, our findings here contrasts with our previous multiclass ROC evaluation to classify disease based on at-arrival differential expression of *ALOX15*, *CFB*, *MARCO*, *LOC100335828*, and *SLC18A2* [31]. While we previously found good to excellent discrimination of Healthy versus Treated_2+ cattle based on the expression levels of single genes in cattle from two independent populations [31], the AUCs resulting from ROC curve analysis in this study were considerably lower when results from all seven populations of cattle were assessed together. For example, in our previous assessment of cattle from two populations, the ROC curve based on differential expression of *ALOX15* to distinguish Treated_2+ from Healthy cattle had an AUC of 0.860 [31], while in this study, the AUC was 0.635 (Table 1). The AUCs resulting from ROC curve analysis for each of the seven populations in this study evaluated individually suggests that these molecules may better discriminate BRD in populations with more severe disease, as seen with the DG_17, DG_18, and MH_19 populations compared to VD_17 and PS_19 (Supplemental Tables S1 and S9). For example, in the DG_17, DG_18, and MH_19 populations, the AUCs for the ROC curves differentiating Treated_2+ from Healthy cattle by differential expression of *ALOX15* were 0.806, 0.760, and 0.800, respectively; compared to VD_17 and PS_19, for which the AUCs were 0.632 and 0.529, respectively (Supplemental Tables S1 and S9).

Corresponding with our differential expression and hierarchical clustering analyses, Treated_2+ cattle were the most dissimilar and routinely possessed the highest AUC values for genes independently evaluated, when compared to Healthy. Given this, we employed a multivariable ROC curve model for the two most explainable mechanisms (IFN and SPM; Table 1) and developed an expressional decision tree model using the ROC-generated cutoffs from Treated_2+ and Healthy comparisons (Fig. 5). While the combination of *ALOX15* and *HPGD* (SPM) made no discernable difference in performance as compared to evaluation of each gene independently, combining the four IFN genes increased the single-test sensitivity and specificity between Treated_2+ and Healthy cattle. The resulting decision tree possessed relatively low accuracy for discerning Healthy from BRD (51.3%), but possessed excellent accuracy in Treated_2+ categorization (90.0%). While Treated_1 individuals are similar to Healthy in terms of at-arrival gene expression, classificational accuracy (68.5%) is moderately higher than the estimated sensitivity of diagnosing clinical BRD via visual assessment alone (22.0–62.0%) [12, 13]. Furthermore, Treated_1 cattle misclassified as not requiring treatment (Healthy) were sold having higher total average daily gains compared to those classified correctly; this may indicate the ability for this decision tree model to better stratify for disease severity compared to visual assessment and treatment frequency associated with BRD.

This report represents our third evaluation of differential gene expression in whole blood at arrival to predict subsequent BRD treatment in cattle, with progressively larger numbers of cattle from more groups evaluated in each study. Genes that were strongly differentially expressed between Healthy and BRD cattle in our earlier work were not always significantly differentially expressed in subsequent studies. Given the heterogeneity of the cattle within and between these populations, and likely variation in the time of sample collection relative to disease onset, perhaps this finding is no surprise. However, some genes, such as *ALOX15* and *CFB*, have been consistently differentially expressed between Healthy and BRD cattle across our studies, suggesting that further evaluation of these molecules and related pathways may improve prediction and understanding of BRD risk. The influence of the imprecise nature of current BRD assessment systems must also be acknowledged, especially when considering individuals without more definitive clinical endpoints such as mortality. The development of a more accurate method of BRD diagnosis is essential to support efforts to accurately predict the disease.

## Conclusions

We sought to evaluate and corroborate the at-arrival expression patterns of mRNA previously identified as differentially expressed between cattle subsequently treated for BRD and cattle not treated. Here, we profiled at-arrival whole blood samples of 234 cattle across seven independent populations via NanoString nCounter and nSolver analysis. At arrival expression levels of mRNA associated with specialized pro-resolving mediator metabolism, anti-inflammatory/apoptotic processes, immune function, and antimicrobial peptide production were increased in cattle never requiring antimicrobial therapy for BRD. Cattle requiring antimicrobial therapy for BRD within 28 days of arrival, especially those needing two or more treatments, possessed increased expression of complement factor B, pro-inflammatory, and type I interferon-associated mRNA. We further evaluated these mRNA expression levels for individual classificational accuracy through receiver operator characteristic curves and the development of a decision tree model. Cattle that would require multiple antimicrobial therapies for BRD were classified with 90.0% accuracy. These findings serve as a foundation for developing an at-arrival mRNA prediction model in high-risk populations of post-weaned beef cattle. As this study assessed at-arrival expression levels, future studies evaluating expression longitudinally are necessary to infer relationships between gene expression and long-term protection against or facilitation of BRD.

## Supplementary Information

**Additional file 1: Table S1.** Metadata of cattle selected for NanoString nCounter mRNA profiling. **Table S2.** Quality control information from sample preparation and RNA isolation. **Table S3.** mRNA probe selection information. **Table S4.** Differentially expressed genes identified between Healthy vs BRD cattle (fold change directionality in Healthy). **Table S5.** Differentially expressed genes identified between Treated_1 vs Healthy cattle (fold change directionality in Treated_1). **Table S6.** Differentially expressed genes identified between Treated_2+ vs Healthy cattle (fold change directionality in Treated_2+). **Table S7.** Differentially expressed genes identified between Treated_2+ vs Treated_1 cattle (fold change directionality in Treated_2+). **Table S8.** Levene’s test for homogeneity of variance across all 30 DEGs. **Table S9.** Selective multiclass receiver operator characteristic (ROC) curve analysis for each study population.

**Additional file 2: Figure S1.** Boxplots of the log2CPM gene expression levels for all 30 DEGs.

## Data Availability

The data presented in this study are openly available in the National Center for Biotechnology Information Gene Expression Omnibus repository (NCBI-GEO; https://www.ncbi.nlm.nih.gov/geo) under the accession GSE179536. All remaining data generated or analyzed during this study are included in this published article and its additional information files.
