# Supplementary figures and images for: Use of nCounter mRNA profiling to identify at-arrival gene expression patterns for predicting bovine respiratory disease in beef cattle
Source: BMC Vet Res. 2022 Feb 23;18:77. doi: 10.1186/s12917-022-03178-8 (PMC8864212; doi:10.1186/s12917-022-03178-8)

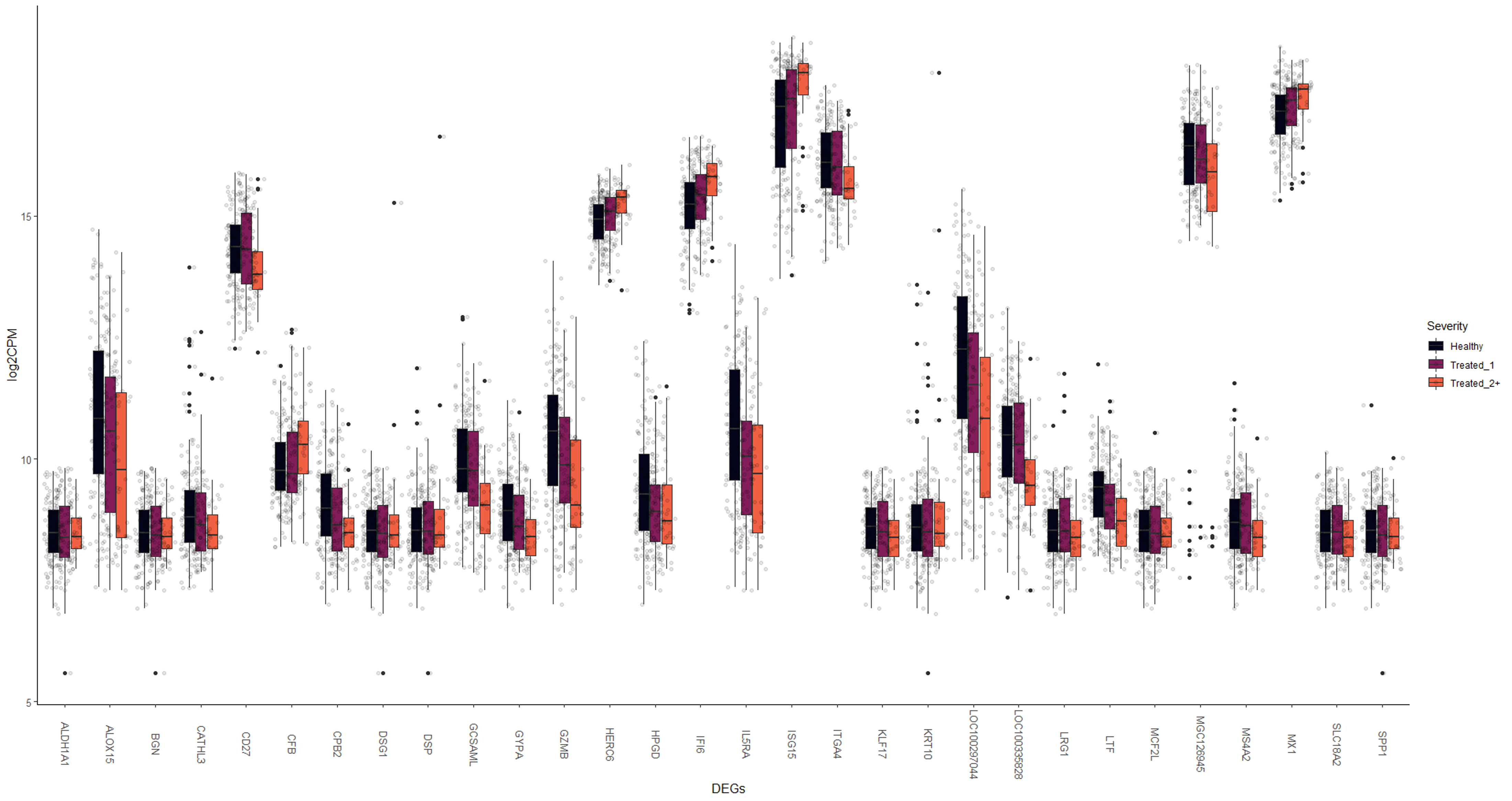

Supplement: Supplementary file 2 — Additional file 2: Figure S1. Boxplots of the log2CPM gene expression levels for all 30 DEGs. [file 12917_2022_3178_MOESM2_ESM.png]
